# Developing low-cost nanohybrids of ZnO nanorods and multi-shaped silver nanoparticles for broadband photodetectors†

**DOI:** 10.1039/d3ra03485b

**Published:** 2023-07-19

**Authors:** Nhat Minh Nguyen, Duc Anh Ngo, Le Ngoc Thu Nguyen, Hoai Nhan Luong, Ha Ngoc Duy Huynh, Bui Gia Man Nguyen, Nhat Giang Doan, Le Thai Duy, Anh Vy Tran, Cong Khanh Tran, Kim Ngoc Pham, Vinh Quang Dang

**Affiliations:** a Faculty of Physics and Engineering Physics, University of Science 227 Nguyen Van Cu Street District 5 Ho Chi Minh City 700000 Vietnam; b Faculty of Materials Science and Technology, University of Science 227 Nguyen Van Cu Street District 5 Ho Chi Minh City 700000 Vietnam vinhquangntmk@gmail.com; c Center for Innovative Materials and Architectures (INOMAR) Ho Chi Minh City 700000 Vietnam; d Vietnam National University Ho Chi Minh City 700000 Vietnam; e Institute of Applied Technology and Sustainable Development, Nguyen Tat Thanh University Ho Chi Minh City 700000 Vietnam; f Faculty of Environmental and Food Engineering, Nguyen Tat Thanh University Ho Chi Minh City 700000 Vietnam

## Abstract

Photodetectors are essential elements for various applications like fiber optic communication systems, biomedical imaging, and so on. Thus, improving the performance and reducing the material costs of photodetectors would act as a motivation toward the future advancement of those applications. This study introduces the development of a nanohybrid of zinc oxide nanorods (ZnONRs) and multi-shaped silver nanoparticles MAgNPs through a simple solution process; in which ZnONRs are hybridized with MAgNPs to enable visible absorption through the surface plasmon resonance (SPR) effect. The photodetector based on ZnONRs/MAgNPs is responsive to visible light with representative wavelengths of 395, 464, 532 and 640 nm, and it exhibits high responsivity (*R*), photoconductive gain (*G*) and detectivity (*D*). The maximum *R* is calculated from the fitting curve of the responsivity-power relation with the value of 5.35 × 10^3^ (mA W^−1^) at 395 nm excitation. The highest *G* and *D* reach 8.984 and 3.71 × 10^10^ Jones at that wavelength. This reveals the promise of our innovative broadband photodetector for practical usage.

## Abstract 2

This study reports the fabrication of a broadband photodetector based on a ZnONRs/MAgNPs hybrid structure with high performance.

## Graphical abstract

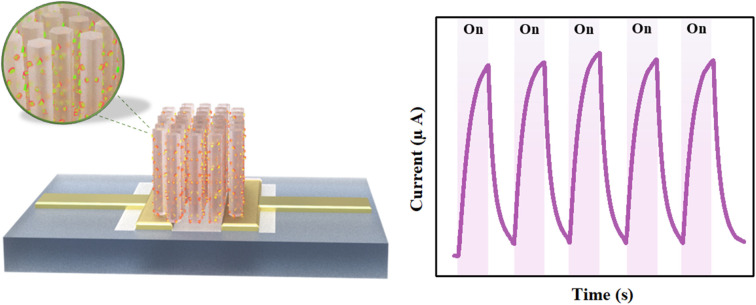

## 1 Introduction

Photodetectors (PDs) play essential roles in almost every aspect of human life such as industry, the military, imaging, communication, *etc.*; therefore, studies into this field have continuously attracted scientists over the years.^1–3^ These devices, operating mainly on the principle of light detection in suitable regions, can be classified into several groups: ultraviolet (UV), visible (Vis), infrared (IR) and sometimes broadband (UV to Vis, Vis to IR, even UV to IR) depending on the optical properties of their active materials.^4^ For instance, while β-Ga_2_O_3_ is appropriate for UV PDs, as reported by the group of J. Yu; (In,Ga)N nanowires were exploited to fabricate a device used in the visible region.^5,6^ Otherwise, Zeng and partners demonstrated that epitaxial-growth PtTe_2_-based PD had the ability to detect light in the IR region with the value of wavelength up to 10.6 μm.^7^ However, researchers are now moving towards broadband phototodetectors, which can operate in a wide range of wavelengths, in order to take advantage of various components in the solar spectrum. In 2021, a two-dimensional layered Ta_2_NiSe_5_ photodetector was reported by Y. Zhang *et al.*, showed a noticeably high performance with the responsivity of 198.1 A W^−1^.^8^ Around the world, this type of device has been investigated so far by many groups, indicating the high demand for a device that is sensitive to different light sources.^9–11^

In terms of photodetector fabrication, various low-dimensional (0D, 1D, 2D) materials can be applied.^12^ Among them, 1D metal oxides (CeO_2_, Cu_2_O, SnO_2_, …) with unique morphologies own superior sensitivity to light thus making this type of material prevailing in photodetectors fabrication.^13^ Up to now, 1D zinc oxide (ZnO), particularly ZnO nanorods (NRs), has been investigated deeply because of their outstanding properties such as durability, large exciton energy of 60 meV and simple synthesis processes.^14–16^ Moreover, the morphology and orientation of ZnONRs (1D), which have a big impact on the performance of the ZnO-based photodetectors could be easily controlled by changing the preparation condition.^17^ However, ZnONRs have never been a suitable material for broadband detection due to several problems, especially the large band gap of 3.3 eV, which means that devices based on this material can operate only in the UV region that accounts for only 4% in the solar spectrum.^18–20^ Therefore, modifying ZnONRs to improve their optical properties has become the interest of research for many years.^21,22^

For years, scientists have sought for solutions to overcome the limitations of ZnO's absorption, and several modification methods as doping with transition metals, decorating with noble metals have been intensively surveyed.^23–26^ The former method utilized the transition metals element consisting of Cu, Ti, Co, Mn, *etc.* on the platform of replacement of the host with the dopant metal atoms.^27^ For instance, by doping copper into ZnO lattice, energy levels of Cu^+^ and Cu^2+^ localize inside the band gap of the host material, leading to the narrow optical band gap.^18,28^ For the latter, metal nanoparticles loaded onto ZnO provide the resonant oscillation of electron clouds under visible excitation, known as surface plasmon resonance (SPR) effect, can effectively contribute to the visible detection of the photodetectors.^29^ Both these two modification approaches have been widely investigated by scientists and significantly enhancement in performance of ZnO-based photodetectors were reported;^30,31^ nevertheless, the discussed methods still have some aspects that need to be improved further. Indeed, although the doping solution has been proven as an effective way to shift the absorption edge to the visible region, some demerits such as long photo-response time and the difficulty in controlling the defects make the industrial manufacture of ZnO-doped photodetectors difficult.^28,32^ Besides, the decoration procedure usually requires state-of-the-art facilities as physical deposition systems, which is high-cost and time-consuming.^33^ Finally, the most problematic challenge is that the modified ZnO-based photodetectors in previous reports were only sensitive to a specific wavelength of just around 400 nm, which is not ideal for the desired broadband devices.^28,29,34^

In this study, for the first time, we employed a solution-processed nanohybrid of ZnONRs and multi-shape silver nanoparticles (MAgNPs) as the active channel of a resistive-type photodetector and reported the device's outstanding response towards a wide wavelengths. Through various characterizations and measurements, our photodetector is confirmed sensitive to light with various wavelengths, including purple (395 nm), blue (464 nm), green (532 nm) and red (640 nm) with relatively good responsivity of 5.35 × 10^3^, 9.84 × 10^2^, 5.0 × 10^2^ and 5.92 mA W^−1^, respectively. Our simple and low-cost process can be applied in the manufacture of ZnO-based broadband PDs and enable a new domain of optoelectronic devices operating in a diversity of wavelengths.

## 2 Experimental

### 2.1. Materials

The chemicals used in this study include zinc oxide nanoparticles 40 wt% dispersion in ethanol (ZnO NPs, 99%, Sigma-Aldrich), zinc nitrate hexahydrate (Zn(NO_3_)_2_·6H_2_O, 99%, Sigma-Aldrich), hexamethylenetetramine (HMTA, ((CH_2_)_6_N_4_)_2_), 99%, Sigma-Aldrich) silver nitrate (AgNO_3_, 99%), trisodium citrate dihydrate ((Na_3_C_6_H_5_O_7_·2H_2_O), 99% Sigma-Aldrich), sodium borohydride (NaBH_4_, 98.0%, Scharlau, Spain), hydrogen peroxide (H_2_O_2_, 30%, Sigma-Aldrich).

### 2.2. Methods

The hydrothermal synthesis method of ZnONRs in this study followed the reported work.^18^ At first, a seed layer of ZnO NPs (dispersed in ethanol) was spin-coated onto a glass substrate at the rate of 3000 rpm for 30 seconds, followed by a heat treatment at 95 °C for 1 hour in order to evaporate the solvent. Next, ZnONRs were hydrothermally grown on the prepared substrates at 95 °C for 3 hours with the nutrient solution containing 50 mM of Zn(NO_3_)_2_·6H_2_O and 50 mM of HMTA. After gently rinsing with water and drying by N_2_, decorating MAgNPs on the ZnONRs sample was carried out by a photoreduction procedure. Particularly, the ZnONRs sample was immersed in the MAgNPs solution and then irradiated toward ultraviolet light for 1 hour and then dried at 40 °C. Here, the synthesis process of MAgNPs was described in Fig. S1 (ESI†).

### 2.3. Device fabrication and characterizations

At first, silver electrodes were patterned on glass substrate by a sputtering process using a shadow mask. Then, a layer of ZnO NPs seed solution was formed on active channel with the area of 0.6 mm^2^, followed by the growth of ZnONRs through a hydrothermal step. Finally, the photodetector was completed by immersing the as-grown ZnONRs substrate in the MAgNPs solution under UV irradiation to decorate the nanoparticles onto the nanorods. The crystal structure of ZnONRs and ZnONRs/MAgNPs was recorded by X-ray diffraction (XRD) spectroscopy performed on the D8 Advance-Bruker diffractometer with the monochromatic Cu-Kα radiation (*λ* = 1.54 Å). The surface morphology and elemental composition (EDX) of the as-synthesized samples were examined using a field emission scanning electron microscope (Model JSM-6500F, JEOL Co. Ltd). The shape of MAgNPs were investigated by a transmission electron microscopy system (TEM, JEOL, JEM-1400) while their size are evaluated through hydrodynamic size by a nanoparticle analyzer (HORIBA, SZ-100). The optical properties were measured through Ultraviolet-Visible (UV-Vis) spectrophotometer (JASCO V670). In terms of the photodetector, the device's performance was assessed through the current – voltage (*I*–*V*) relation and current depending on time (*I*–*t*) curves which were recorded by the system Keithley 2400.

## 3 Results and discussion

### 3.1. Structural properties

**Fig. 1:**
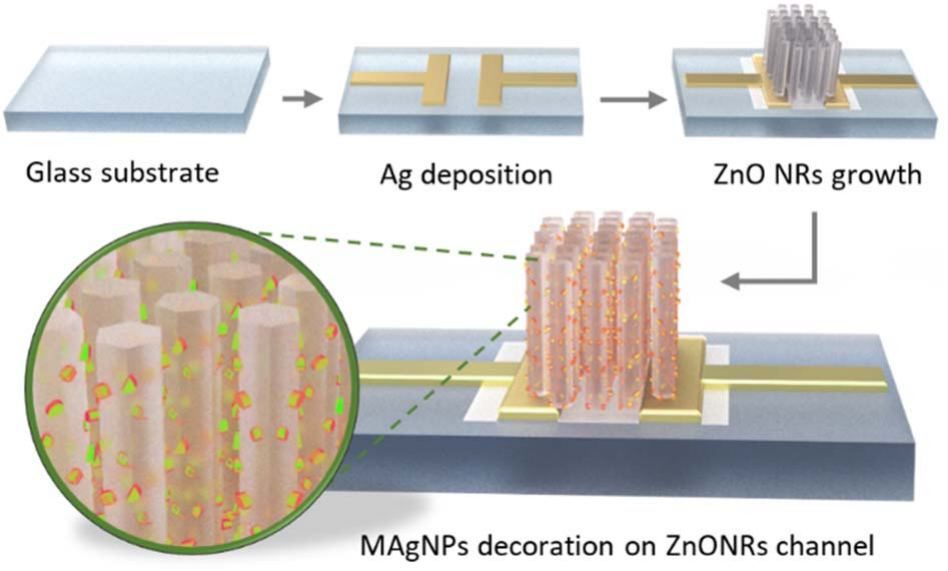
Device fabrication process.

Fig. 1 illustrates the fabrication process of our photodetector. Briefly, after deposition of silver electrodes, the coating of ZnO NPs (as a seed layer) and the hydrothermal growth of ZnONRs were carried out. Finally, the fabrication of a ZnONRs/MAgNPs photodetector was completed by decorating MAgNPs onto ZnONRs through a photoreduction process.

**Fig. 2:**
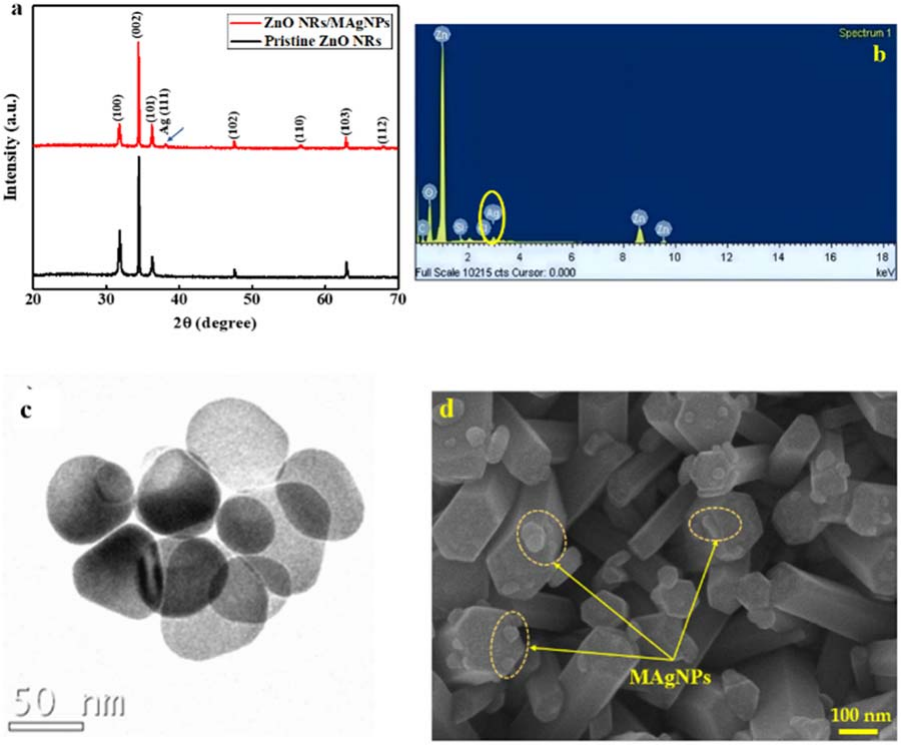
(a) XRD patterns of pristine ZnONRs and ZnONRs/MAgNPs. (b) EDX spectrum of ZnONRs/MAgNPs. (c) TEM image of MAgNPs and (d) FE-SEM image of the nanohybrid channel based on ZnONRs and MAgNPs.

The XRD patterns of the ZnONRs and ZnONRs/MAgNPs samples are presented in Fig. 2a. In general, both samples exhibit the typical diffraction peaks at 2*θ* = 31.73°, 34.43°, 36.25°, 47.61°, 56.67°, 62.85° and 67.93° which correspond to the (100), (002), (101), (102), (110), (103), and (112) planes of ZnO hexagonal structure. The highest intensity of the (002) peak indicates the preferred orientation along *c*-axis of the as-synthesized ZnONRs. This is consistent with the referent data from JCPDS card no. 36-1451 and previously reported studies.^35,36^ Noticeably, we found a small peak at 2*θ* = 38.09° appeared in the XRD pattern of the ZnONRs/MAgNPs sample, which can be attributed to the (111) plane of Ag.^37,38^ Here, a small signal of Ag in the EDX spectrum (Fig. 2b) confirmed the existence of MAgNPs in the ZnONRs/MAgNPs sample (without Ag electrodes). Besides, a small loading of these nanoparticles onto ZnONRs does not affect the structure of the host material as the XRD peaks remain unchanged.

Regarding the materials morphologies, TEM image of MAgNPs (Fig. 2c) shows various shapes of the Ag nanoparticles ranging from circle to triangle, oval with the sizes of several dozens of nanometers. The diversity in shape and size of MAgNPs may be the element contributing to the visible absorption enhancement of the ZnONRs/MAgNPs hybrid structure, which will be discussed later. The surface morphology of the hybrid sample is shown by FE-SEM image in Fig. 2d. Overall, the ZnONRs were grown into a hexagonal structure with high density. Around the nanorods, there are some particles attributed to MAgNPs. Along with XRD and EDX results, this FE-SEM image once again confirms the successful decoration of MAgNPs onto the ZnONRs.

### 3.2. Optical properties

**Fig. 3:**
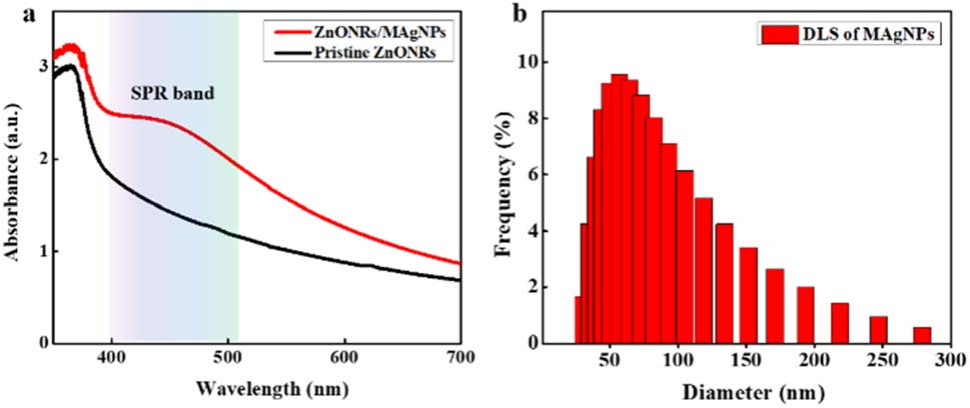
(a) UV-vis absorption spectra of the pristine ZnONRs and ZnONRs/MAgNPs samples and (b) DLS analysis of MAgNPs.

Fig. 3a shows the UV-vis absorption spectra of a pristine ZnONRs sample and a nanohybrid ZnONRs/MAgNPs sample. It can be clearly observed that both samples demonstrate absorption peaks at nearly 368 nm due to the excitonic absorption of ZnO.^39,40^ It can be clearly observed that both samples demonstrate absorption peaks at nearly 368 nm due to the excitonic absorption peaks of ZnO. For pristine ZnO, an absorption in visible light region is detected, which may be a result of band tail formation inside the ZnONRs during the synthesis process;^41,42^ however, this absorption is small in comparison with that of ZnONRs/MAgNPs sample. Furthermore, in the ZnONRs/MAgNPs sample, a wide absorption region ranging from just under 400 to more than 500 nm is determined, which is assigned to the surface plasmon resonance (SPR) effect of MagNPs.^43,44^ In fact, since the sizes of the silver nanoparticles have a certain effect on the SPR wavelengths, wide SPR band can be explained by the size diversity of the MAgNPs which is confirmed by dynamic light scattering (DLS) analysis in Fig. 3b.^45,46^ Therefore, TEM, SEM images (Fig. 2c and d) and UV-vis, DLS spectra (Fig. 3a and b) confirm the successful synthesis and the decoration of MAgNPs onto ZnONRs. Due to the appropriate wide absorption, the hybrid structure ZnONRs/MAgNPs was chosen to fabricate broadband photodetector and 395, 464, 532, 640 nm light was employed as excitation sources to evaluate its performance.

### 3.3. Photodetector characteristics

**Fig. 4:**
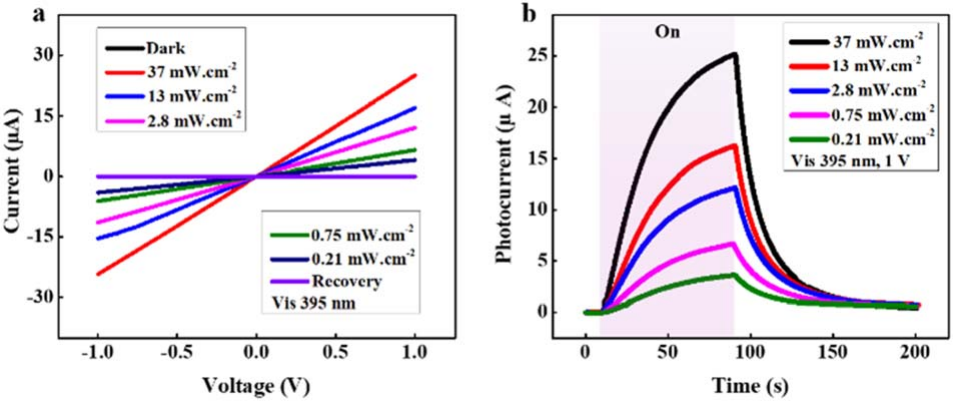
(a) *I*–*V* characteristics and (b) time-dependent photocurrent measurements of the ZnONRs/MAgNPs hybrid photodetector under exposure to various intensities of 395 nm light.

*I*–*V* characteristics of the photodetector under 395 nm-light exposure at different intensities (*P*) are measured and presented in Fig. 4a. Here, the linear *I*–*V* relations with high symmetry under forward and reverse bias reveal the good ohmic metal-semiconductor contact.^47^ Accordingly, as the light intensity rises, the current goes up remarkably and the ZnONRs/MAgNPs photodetector exhibits a comparatively good on/off ratio of 1.744 × 10^3^ at *P* = 37 mW cm^−2^ under 1 V bias. Especially, there is almost no disparity between the dark and the recovery lines in the *I*–*V* characteristics, meaning that the device possesses stable performance.

To have a profound inside into the hybrid photodetector operation, time-dependent photocurrent measurements (*I*_ph_–*t*), in which *I*_ph_ = *I*_light_ − *I*_dark_,^48^ are taken into consideration. The photocurrent data at a constant bias of 1 V is shown in Fig. 4b. Clearly, the current rises when the 395 nm light is turned on and reduces when the light is turned off. Especially, the photocurrent climbs with the increase of light intensity, explained by the relationship between *I*_ph_ and *P* according to the formula *I*_ph_ = *A* × *P*^*θ*^,^49^ in which *A* stands for the wavelength constant and *θ* represents the exponential number. The recorded photocurrents match well with the *I*–*V* characteristics as presented previously.

Regarding the device's response time, it is determined as the time for the photocurrent through the photodetector to reach 63% its maximum and recovery time, defined as the time to return back *ca.* 37% its highest value.^50,51^ Here, we found that the response time and recovery time were about 27.04 and 15.81 seconds, respectively, toward the 395 nm-light exposure. Although the response time and recovery time are still comparatively long, they are still shorter than that of some ZnO-based photodetectors,^29,52,53^ indicating the potential of using our nanohybrid device for practical applications.

Other crucial parameters of a photodetector as responsivity (*R*), photoconductive gain (*G*) and detectivity (*D*) are calculated to evaluate the device's performance. First, *R* is defined as the ratio between the generated photocurrent and the incident light intensity, as described by:
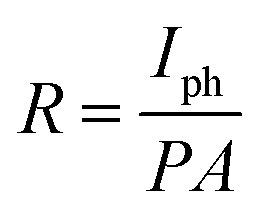


Then, the photoconductive gain, determined as the number of carriers detected per an absorbed photon can be obtained by applying the equation:
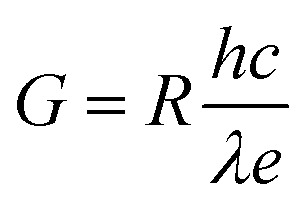
And finally, detectivity that represent the ability to detect weak signals of light, is assessed by:
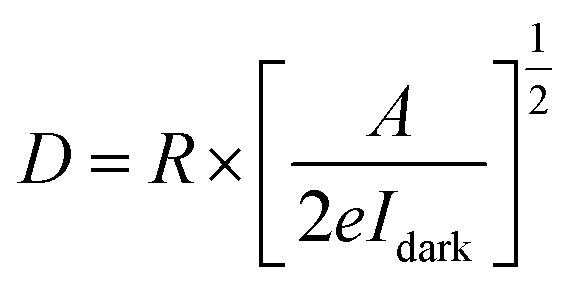
where *I*_ph_, *P* and *A* are the photocurrent, the incident light intensity and the effective device area (0.6 mm^2^) in the given order, *h* stands for the Planck's constant, *c* represents the velocity of light, *λ* and *e* are the wavelength and the electron charge, respectively.^54–56^

**Fig. 5:**
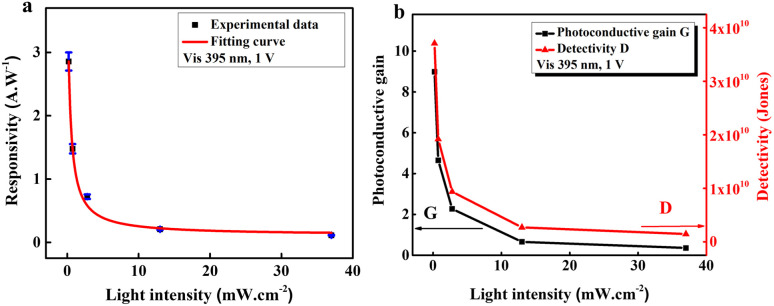
(a) Responsivity as a function of *P* with the fitting curve and (b) photoconductive gain and detectivity of the photodetector *versus P*.

According to the mentioned platform, *R*, *G*, *D* of the ZnONRs/MAgNPs device under 395 nm-light exposure are measured and presented in Fig. 5. It can be seen in this figure that *R*, *G* and *D* decline as the light intensity goes up. Particularly in Fig. 5a, the collected experimental data of *R* fitted relatively well with the function 
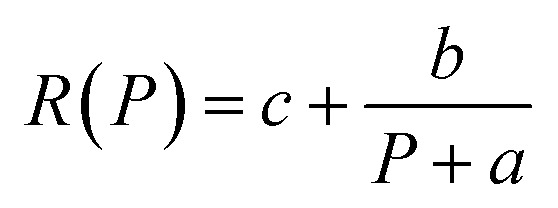
; therefore, the maximum *R* achieved at very low excitation power (*P* → 0) is *ca.* 5.35 A W^−1^. Besides, Fig. 5b indicates that the highest recorded value of *G* and *D* under the same 395 nm-light exposure are 8.98 and 3.71 × 10^10^ Jones, respectively.

Interestingly, similar behaviors in both *I*–*V* and *I*_ph_–*t* relations were also observed when the photodetector was exposed to other wavelengths including 464 nm (Fig. S2a and b†), 532 nm (Fig. S2c and d†) and 640 nm (Fig. S2e and f†). Besides, photocurrent of both pristine ZnONRs and ZnONRs/MAgNPs photodetectors were measured and plotted *versus* wavelengths (Fig. S3†). Clearly, pristine ZnONRs device does not exhibit response towards light in invisible region, except a small rise in photocurrent under 395 nm illumination, which may be the result of band tail formation.^41,42^ However, this value is trivial compared with that of ZnONRs/MAgNPs device. The response and recovery times of the device toward each wavelength are evaluated and listed in Table S1.† The *R*, *G*, and *D* values as a function of *P* toward each wavelength can be assessed through Fig. S4.† Clearly, the values of *R*, *G*, and *D* toward 464 nm or 532 nm wavelengths (Fig. S4a and d†) slightly decreased when *P* increased but its *R* values was also fit with the same function as exposed toward 395 nm light. However, when excited by the 640 nm light source, the device witnesses a rise in all three mentioned parameters at the high intensity (Fig. S4e and f†). We assumed that this was partly due to the thermal effect, which arose under the exposure condition of long wavelength (640 nm) at high intensity and contributes to the generation of charge carriers. Thus, the highest *R* of our photodetector under this wavelength is reported at *P* = 1.65 mW cm^−2^.

**Fig. 6:**
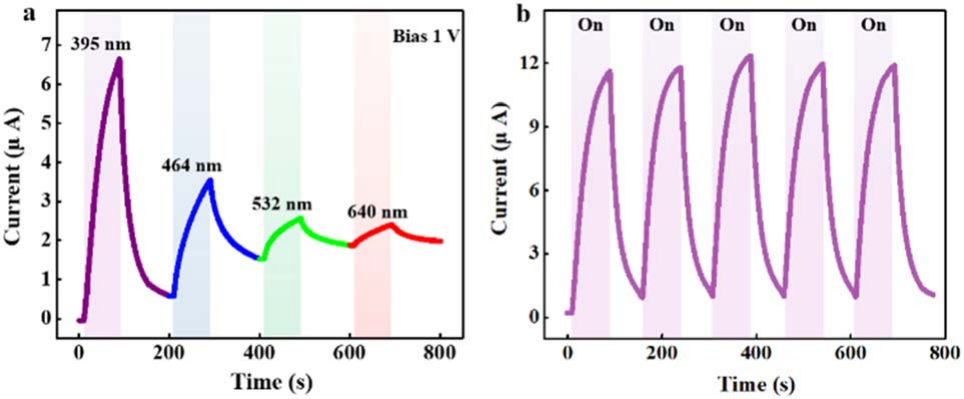
(a) Time-dependent current measurement of the device under four light sources with specific wavelengths of 395, 464, 532, and 640 nm. (b) Cyclic exposure test (5 cycles) toward 395 nm light.

Broadband photodetection property of our hybrid device is demonstrated in Fig. 6a. Herein, under several excited wavelengths, the values of the photocurrent climb, revealing the sensitivity of wide-range excitations. The photodetector's stability is also investigated and represented by Fig. 6b. Apparently, under repeated stimulation when the light is continuously turned on and off, the changes in the device's performance are negligible, exhibiting the prospect of long-term operation.

Table 1 shows the summary of the ZnONRs/MAgNPs photodetector and comparison with several studies. Clearly, our device exhibits sensitivity to longer wavelengths under lower bias voltage compared with the others. Furthermore, the photodetector's typical parameters as *R*, *G*, and *D* are comparable to those of other devices reported by other research groups. Although the values of *R*, *G* and *D* obtained in our study are not superior, the simple solution-processed ZnONRs/MAgNPs material is still potential for practical broadband detection applications.

**Table 1:** Comparison of the ZnONRs/MAgNPs hybrid photodetector with some publications

Materials structure	*λ* (nm)	*R* (mA W^−1^)	*G*	*D* (Jones)	Bias (V)	Ref.
ZnONRs	365	8.78 × 10^2^	—	—	5	52
Mn doped ZnO film	365	2.75	—	1.7 × 10^10^	3	57
Al doped ZnO film	385	4.6 × 10^2^	—	1.63 × 10^10^	5	58
Cu-doped ZnONRs/PEDOT:PSS	395	3.3 × 10^2^	—	—	5	28
ZnONRs/AgNPs	380	12.4 × 10^3^	—	2.72 × 10^11^	0.2	59
ZnONRs/AgNPs	400	46	0.101	—	5	29
ZnO/Ag NW/ZnO	365	10^2^	—	6.8 × 10^12^	1	51
AZO/ZnO/PVK/PEDOT:PSS	365	81.6	—	3.5 × 10^9^	−5	60
PVK/ZnONRs/Graphene	365	80.6 × 10^3^		2.3 × 10^11^	5	61
*n*-ZnO NRs/i-MgO/p-GaN	350	3.2 × 10^2^	—	>12 × 10^12^	0	62
2D-MoS_2_/1D-ZnO	365	24.36 × 10^3^	—	—	5	63
	532	3.5 × 10^2^	—	—	5	
ZnONRs/MAgNPs	395	5.35 × 10^3^	8.984	3.7 × 10^10^	1	This work
	464	9.84 × 10^2^	1.727	3.6 × 10^9^		
	532	5.0 × 10^2^	0.142	3.4 × 10^8^		
	640	5.92	0.011	3.3 × 10^7^		

### 3.4. Sensing mechanism

**Fig. 7:**
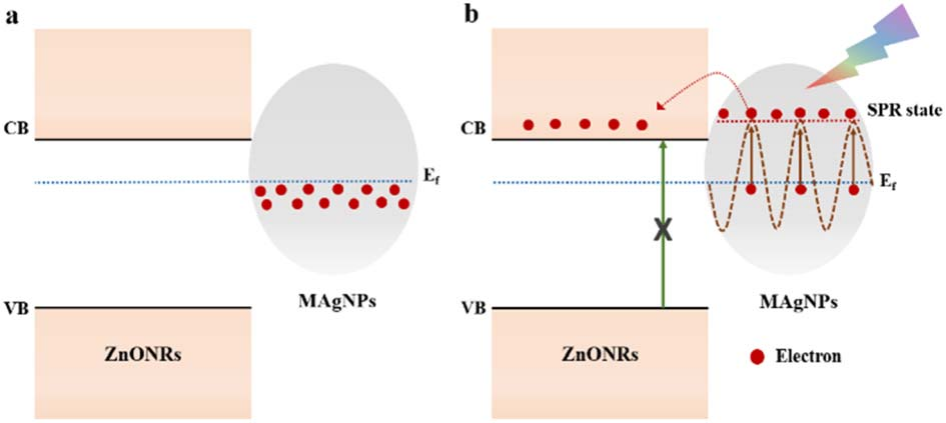
Energy band diagram of the photodetector under (a) dark and (b) light conditions.

Energy band diagrams of the hybrid structure are depicted in Fig. 7. In other words, in dark condition (Fig. 7a), there is a metal-semiconductor contact formed at the interface between ZnONRs and MAgNPs due to the Fermi levels alignment.^51^ Under light exposure (Fig. 7b), SPR effect of MAgNPs occurs. Indeed, the incident photons are absorbed by MAgNPs, which leads to the oscillation of electron clouds with a typical frequency. If the frequency of the excitation light matches the specific frequency of the electron clouds, these clouds undergo resonant oscillation and the electrons inside the MAgNPs become highly energetic, known as “hot electrons”. Consequently, because the excited state of the hot electrons (SPR state) is higher than the conduction band (CB) of ZnONRs, these “hot electrons” will easily move towards the ZnONRs and transfer into the electrodes, generating the photocurrent through the device.^24,29,64,65^ Therefore, the response of ZnONRs/MAgNPs hybrid structure to visible illumination is strongly attributed to the SPR effect occurring in the MAgNPs. Moreover, since it was demonstrated that when the size of silver nanoparticles increases, the SPR wavelength becomes longer,^46^ the usage of MAgNPs in this work plays a significant role. Indeed, because the synthesized MAgNPs possess different shapes (triangle, sphere, oval, *etc.*), which leads to various sizes, the SPR effect can occur in many wavelengths; therefore, broadband response of ZnONRs/MAgNPs photodetectors is observed.

## 4 Conclusions

In summary, a ZnONRs/MAgNPs hybrid broadband photodetector is fabricated by a simple solution procedure. Due to the novel utilization of MAgNPs with different shapes and sizes, the device reveals a noticeable sensitivity to a wide range of wavelengths, including 395 nm, 464 nm, 532 nm and 640 nm with the maximum responsivity of 5.35 × 103, 9.84 × 102, 5.0 × 102, and 5.92 mA W^−1^, respectively. Other device's parameters also exhibit acceptable values, from 0.011 to 8.984 for the photoconductive gain and from 3.3 × 10^7^ (Jones) to 3.71 × 10^10^ (Jones) for the detectivity. This simple hybrid structure is believed to pave the way for studies into a new domain of high-performance broadband photodetector in the future.

## Author contributions

N. M. Nguyen: conceptualized and carried out experiments on ZnONRs. L. N. T. Nguyen, H. N. Luong: carried out experiments on MAgNPs. D. A. Ngo: calculated the photodetector's parameters. H. N. D. Huynh, B. G. M. Nguyen, N. G. Doan: analyzed the XRD, EDX, SEM, TEM results. C. K. Tran: analyzed the UV-vis and DLS results. K. N. Pham and A. V. Tran: measured the photodetector's performance (*I*–*V* and *I*–*t*). L. T. Duy: preparing the original manuscript. V. Q. Dang: monitored the experiments and edited the manuscript.

## Conflicts of interest

There are no conflicts to declare.

## Supplementary Material

RA-013-D3RA03485B-s001
