# Supplementary material for: Developing low-cost nanohybrids of ZnO nanorods and multi-shaped silver nanoparticles for broadband photodetectors
Source: RSC Adv. 2023 Jul 19;13(31):21703–9. doi: 10.1039/d3ra03485b (PMC10354500; doi:10.1039/d3ra03485b)
Supplement: RA-013-D3RA03485B-s001 [file RA-013-D3RA03485B-s001.pdf]

Electronic Supplementary Material (ESI) for RSC Advances.
This journal is © The Royal Society of Chemistry 2023

Supporting information

Developing low-cost nanohybrids of ZnO nanorods and multi-shaped silver nanoparticles for broadband photodetectors

Nhat Minh Nguyen[a,d], Duc Anh Ngo[b,d], Le Ngoc Thu Nguyen[b,d], Hoai Nhan Luong[b,d], Ha Ngoc Duy Huynh[b,d], Bui Gia Man Nguyen[b,d], Nhat Giang Doan[b,d], Le Thai Duy[b,d], Anh Vy Tran[e,f], Cong Khanh Tran[b,d], Kim Ngoc Pham[b,c,d], and Vinh Quang Dang[b,c,d,*]

[a] Faculty of Physics and Engineering Physics, University of Science, 227 Nguyen Van Cu Street, District 5, Ho Chi Minh City 700000, Vietnam

[b] Faculty of Materials Science and Technology, University of Science, 227 Nguyen Van Cu Street, District 5, Ho Chi Minh City 700000, Vietnam

[c] Center for Innovative Materials and Architectures (INOMAR), Ho Chi Minh City 700000, Vietnam

[d] Vietnam National University, Ho Chi Minh City (VNU-HCM) 700000, Vietnam

[e] Institute of Applied Technology and Sustainable Development, Nguyen Tat Thanh University, Ho Chi Minh City 700000, Vietnam

[f] Faculty of Environmental and Food Engineering, Nguyen Tat Thanh University, Ho Chi Minh City 700000, Vietnam

* Corresponding e-mail: vinhquangntmk@gmail.com

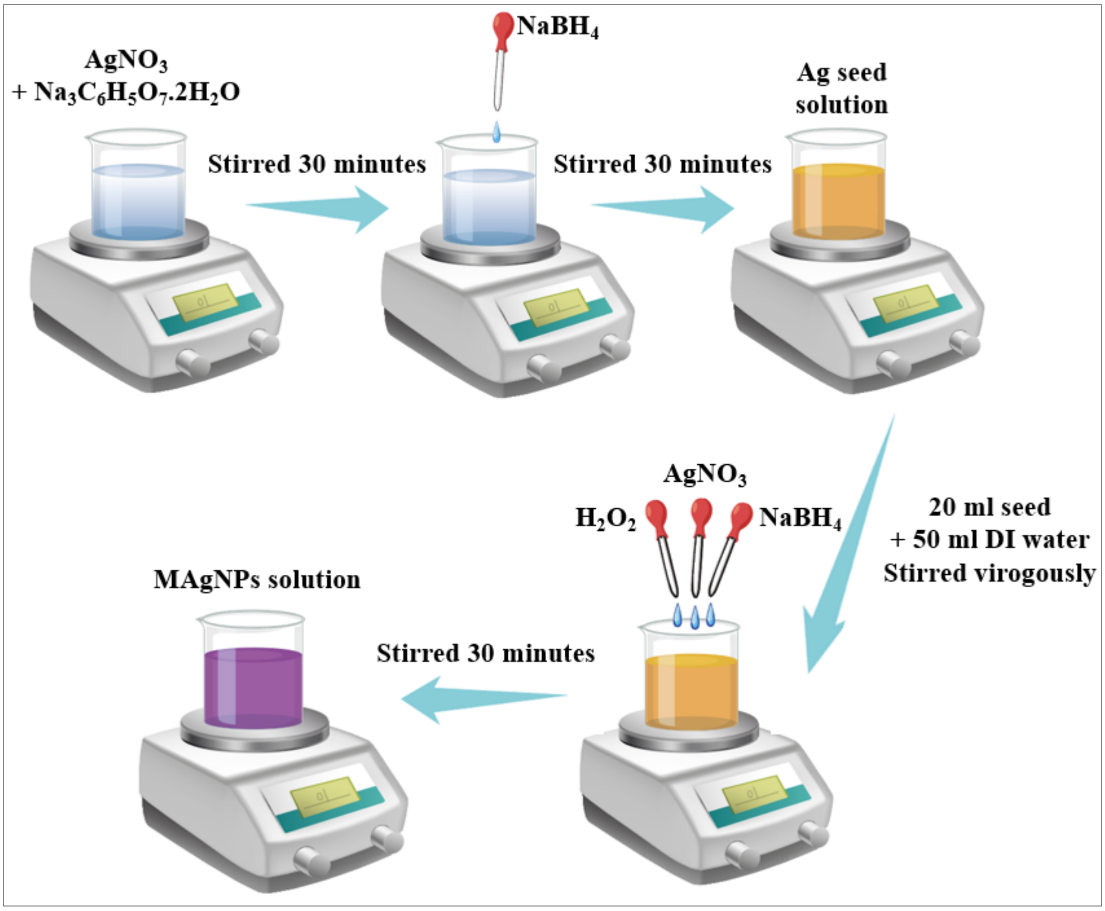

Figure S1. The synsthesis process of MAgNPs solution

The formation of MAgNPs solution is a result of an etching-growth process, as indicated by our group [1,2]. Initially, the Ag seed solution was synthesized by mixing 0.25 mM $AgNO_3$ with 0.25 mM $Na_3C_6H_5O_7.2H_2O$ under stirring condition for 30 minutes, followed by a slow dropping procedure of 10 mM $NaBH_4$. Consequently, to obtain MAgNPs, 20 mL of Ag seed was mixed with 50 mL DI water. Then, after adding 8 % H_2O_2 to the being-stirred beaker, 20 mM $AgNO_3$ and 52.87 mM $NaBH_4$ were simultaneously poured into the solution. The final purple solution was consistently stirred for further 30 minutes to stabilize the MAgNPs.

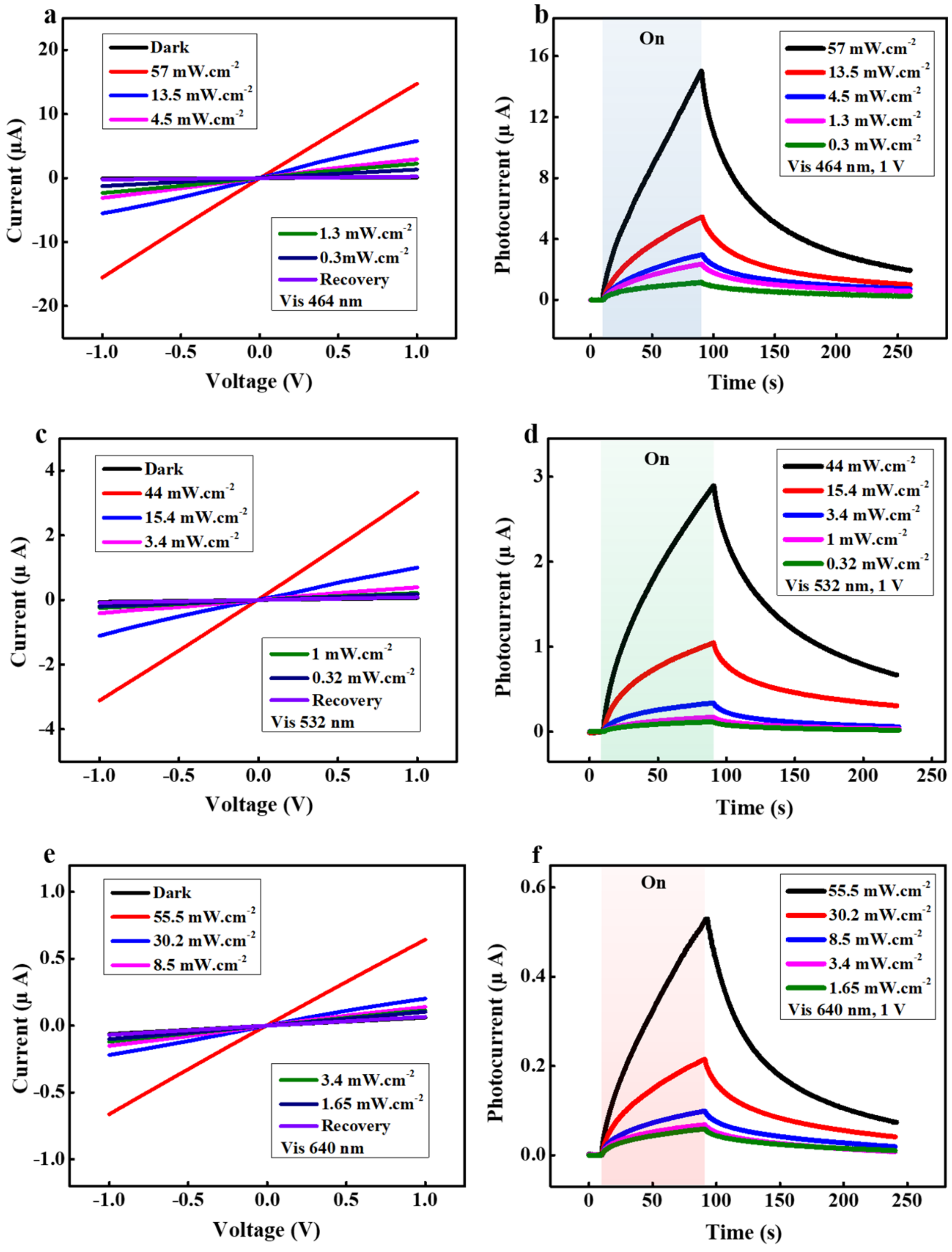

Figure S2. I-V characteristics and time-resolved photocurrents of the photodetector under various excitation light wavelengths at 464 nm (**a** and **b**), 532 nm (**c** and **d**), 640 nm (**e** and **f**).

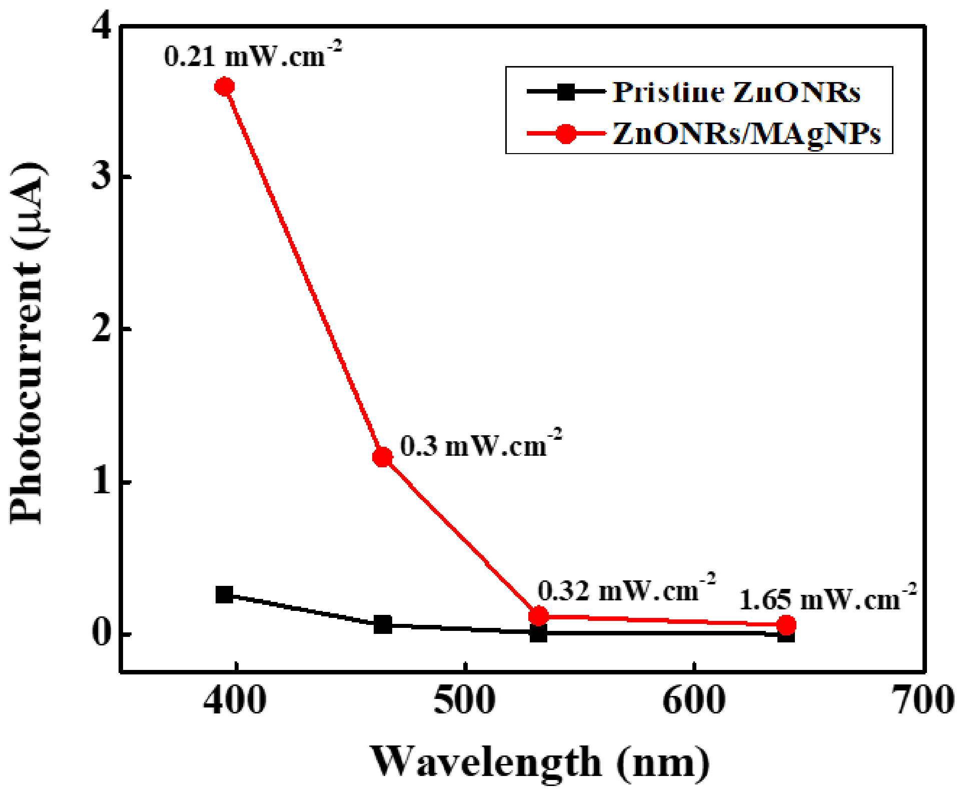

Figure S3. Photocurrent of ZnONRs and ZnONRs/MAgNPs photodetectors as fucntions of wavelength

Table S1. Summary of calculated response and recovery times of the photodetector under 464, 532 and 640 nm illumination

**Wave length (nm)**	**464**	**532**	**640**
**Response time (s)**	40.54	29.83	28.658
**Recovery time (s)**	61.509	48.94	63.115

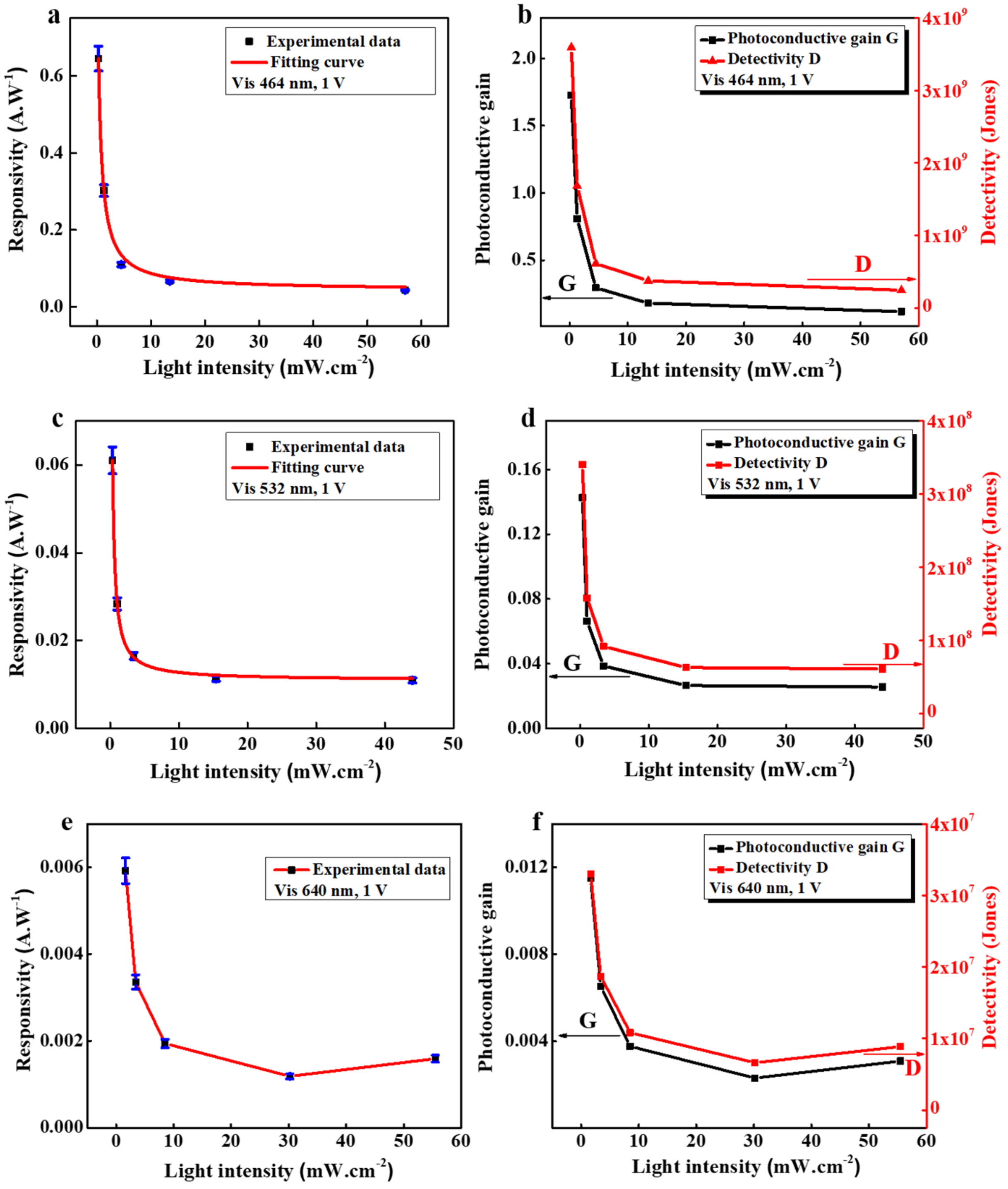

Figure S4. Dependence of responsivity, photoconductive gain and detectivity of the photodetector on light intensity at 464 nm (**a** and **b**), 532 nm (**c** and **d**), 640 nm (**e** and **f**).

References

[1] Pham T B N, Bui T T T, Tran V Q, Dang V Q, Hoang L N and Tran C K 2020 Surface-enhanced Raman scattering (SERS) performance on salbutamol detection of colloidal multi-shaped silver nanoparticles *Appl Nanosci* **10** 703–14

[2] Luong H N, Nguyen N M, Nguyen L N T, Tran C K, Nguyen T T, Duy L T, Nguyen N P, Huynh T M H, Tran T T, Phan B T, Thi T V T and Dang V Q 2022 Detection of carbendazim by utilizing multi-shaped Ag NPs decorated ZnO NRs on patterned stretchable substrate through surface-enhanced Raman scattering effect *Sensors and Actuators A: Physical* **346** 113816
